# A Case of Hepatitis; with Remarks

**Published:** 1789

**Authors:** George Wilkinson

**Affiliations:** Surgeon at Sunderland, Member of the Royal College of Surgeons, and Honorary Member of the Chirurgo-Physical Society of Edinburgh


II. *A Case of Hepatitis; with Remarks. Communicated in a Letter to Dr.* Simmons, *F. R. S. by Mr.* George Wilkinfon, *Surgeon at Sunderland, Member of the Royal College of Surgeons, and Honorary Member of the Chirurgo-Phyfical Society of Edinburgh.*

JOHN KEMP, a Schoolmafter, aged fixty-five years, and of a pretty hale conftitution, was feized in June, 1783, with a fhivering fit, fucceeded by cough, dyfpnœa, flight expectoration, and a heavy pricking pain, as he defcribed it, at the lower part of the fternum, particularly towards the right fide. His pulfe was fuller and quicker than ufual, but he had no great thirft: his appetite was diminifhed, and he was coftive.

To relieve thefe fymptoms volatile liniment was applied to the part affected, and a blifter between the fhoulders. A purgative was adminiftered occafionally, and he was directed to take the lac ammoniaci with oxymel of fquills in the courfe of the day, and a mild opiate at bed time.

Under this treatment he foon grew better, fo that at the end of eight or nine days I difcontinued nued my visits, and saw no more of him till September 12th, when I was again called to his assistance. I then found him labouring under a great difficulty of breathing, (though sitting up in a chair) with a yellowness of the eyes, and his legs in a very œdematous state. He complained much of thirst, and had frequent inclination to vomit; his pulse was very quick; his urine remarkably high coloured, and little in quantity, and at this time his belly was so obstinately constipated, that he had passed no stool for nine days.

His complaint, which, at the time of my former attendance, I had suspected to be pulmonary, and treated accordingly, now bore evident marks of being a case of hepatitis; and of this I was more clearly convinced on perceiving a pretty large and somewhat unequal swelling extending from under the xiphoid cartilage to the navel, and which seemed to elevate a little the false ribs of the right side.

Upon my inquiring more particularly into the cause and progress of his complaint, the patient informed me he had often felt a sort of uneasiness or pain about the region of the liver, which he imagined might proceed from his leaning much against a desk in writing. This pain, he obferved, had gradually increafed for fome time before I firft faw him, which was now about three months fince; and he had been attacked with feveral irregular fhivering fits towards night, accompanied with fweating, and had been much troubled with coftivenefs. The colour of his ftools had for fome time been whitifh.

The region of the liver was now not only extremely painful on the flighteft preffure, but the patient likewife felt the pain extend to the top of his fhoulder on the right fide, more particularly whenever he attempted to lie or turn on the oppofite fide.

The nature of the difeafe being now fo fully afcertained, I determined (notwithftanding the advanced age of the patient and the great reafon I had to fear that a fuppuration had already taken place) to try the effect of mercury, bearing in mind the adage of Celfus, *melius eft anceps remedium quam nullum.*

I began by adminiftering a purgative clyfter, and in the courfe of the day he took two fcruples of *pilul. ex colocynth.* and fix grains of calomel made into pills. Thefe medicines, however, had produced no evacuation on the morning of the 13th, notwithftanding another clyfter had been injected and all the pills had been retained, tained, though not without ſome difficulty on account of the irritable ſtate of the ſtomach.

Being induced to think that a ſuperabundance of acidity might be the cauſe of hindering the ſolution and conſequent operation of the medicines lately adminiſtered, I directed him to take two ſcruples of ſalt of tartar and half a drachm of magneſia alba in a ſuitable vehicle every two hours. The good effects of this mixture were evidently conſpicuous on his taking the ſecond doſe, as it not only produced a very copious ſtool, but likewiſe a conſiderable diſcharge of urine.

Two drachms of the ſtrong mercurial ointment were now directed to be rubbed on the region of the liver, and five grains of calomel were adminiſtered in a bolus at bed time. Theſe remedies were occaſionally repeated till the 18th. He had then rubbed in about ſix drachms of the ointment, and taken twenty grains of calomel. The ſwelling had greatly ſubſided, and was leſs painful when preſſed; but he complained of a fulneſs about the umbilical, or rather hypogaſtric region, which, on my ſtriking it with my finger, ſhewed evident ſigns of fluctuation. His ſtools hitherto had been whitiſh; but this day he voided one which appeared to be mixed with bile. The day following (Sept. 19) the mercurial treatment was ſuſpended, and, in its ſtead, I directed a ſaline mixture with oxymel of ſquills, aromatic tincture, and laudanum.

His mouth, by this time, was become affected, and a moderate ſalivation had taken place; the yellowneſs of the tunica conjunctiva had diſappeared, and the quantity of his urine was conſiderably increaſed. On the 20th, however, he was ſuddenly alarmed upon voiding, by ſtool, a conſiderable quantity of whitiſh purulent matter, which bore ſome reſemblance to the coagulable lymph that is oftentimes ſeen in the cavity of the abdomen in inflammation of the peritonæum.

I found him very faint after this evacuation, and obſerved, to my great ſurpriſe, that the ſwelling of the belly had entirely ſubſided.

More or leſs of the purulent diſcharge continued to appear with his ſtools till September 27th, at which period he ſeemed to be beginning to recover, though he was ſtill extremely weak. His ſalivation had ceaſed, and his urine was in proper quantity, though ſometimes mixed with a ſort of mucus reſembling pus. I now adviſed advifed him to take a decoction of bark occafionally, and an opiate at bed time.

Two days after, viz. on the 29th, I was alarmed on finding him uneafy and feverifh, with a quick pulfe, and thirft, he having been feized the preceding evening with a fmart fhivering fit, and paffed a very indifferent night.

I now determined to have recourfe immediately to the bark in fubftance, and he took of it from two fcruples to a drachm every three or four hours. This mode of treatment was perfevered in, with fome little variation, for about a fortnight, at the end of which time he was well recovered, and is now in a tolerable ftate of health.

This cafe may perhaps claim the attention of the medical reader on account of its remarkable termination, and of its affording a well-marked inftance of a difeafe that is generally allowed to be of rare occurrence in Europe, I mean confidered as a primary affection, for fuch it feems to have been in my patient. In the fymptomatic or fecondary hepatitis, which is by far the moft frequent fpecies of the difeafe in this country, the complaint rarely terminates in fuppuration, but in a fchirrus, of which the confequence is dropfy.

In the caſe I have related there ſeems to be great reaſon to think that the complaint was a partial one, I mean with reſpect to its being confined to a part of the liver, and to a part that favoured its exit into the inteſtinal canal. This will appear, perhaps, ſtill more probable when we conſider the ſpeedy termination of the diſeaſe, and the facility with which the patient recovered.

It muſt be allowed that abſceſſes of the liver terminate leſs frequently by ſtool than otherwiſe; yet it is perhaps not improbable that they occur much more frequently than medical practitioners, from not always attending ſufficiently to the ſtate of the fæces, are aware of.

What ſhare the uſe of mercury had in the favourable termination of this caſe I will not pretend to determine; but it ſeems to be certain that this remedy has been uſed with a degree of ſucceſs ſuperior to every other in affections of the liver; and the remarkable caſe, recorded by Dr. Clark *, wherein it would ſeem that ſuppuration had begun to take place, induced me, notwithſtanding the diſparity of our patients ages, to venture on its uſe.

* Edin Med. Comm. Vol. V. p. 423.

If

If the nature of this cafe had been clearly afcertained in the beginning, fuppuration might perhaps have been prevented; but the obfcurity of the fymptoms led me, as I have already mentioned, to confider them as effects of pulmonary affection only, and of courfe to lofe fight of the main object. This cafe, therefore, befides ferving as a ftriking proof of the wonderful refources of nature in affections of this kind, may likewife be of ufe with refpect to the diagnoftic in fimilar cafes. Thefe confiderations, Sir, have induced me to communicate it to you for infertion in your valuable publication.

*Sunderland*,

February 10, 1789.

